# A Quadratic Bifactor Hierarchical Model for Jointly Modelling the Nonlinear Relationship Between Response Accuracy and Response Time in Cognitive Testing

**DOI:** 10.3390/jintelligence14080183

**Published:** 2026-08-08

**Authors:** Xiaojun Guo, Xiaohua He, Xiaoyun Bai, Juan Yan

**Affiliations:** 1 School of Education Science, Gannan Normal University, Ganzhou 341000, China; 2 School of Education, Central China Normal University, Wuhan 430079, China

**Keywords:** bifactor model, response time, response accuracy, nonlinear relationship, joint modeling

## Abstract

In computerized assessments, response accuracy and response time together reflect how examinees engage with test items. Existing joint models typically assume a linear relationship between these two outcomes, yet empirical observations often reveal more complex patterns. This study proposes a Quadratic Bifactor Hierarchical Model (QBi-HM) that captures nonlinear speed–accuracy dependencies through a shared general factor with quadratic terms, while preserving separate ability and speed components. Simulation results across sample sizes (*N* = 1000, 1500, 3000) and test lengths (*m* = 30, 60) demonstrated that QBi-HM recovered parameters accurately under both linear and nonlinear conditions, whereas the conventional linear bifactor model produced biased estimates when nonlinearity was present. An empirical application to PISA 2012 computer-based mathematics data (*N* = 1527 examinees from four economies, *m* = 10 items) showed that QBi-HM achieved superior model fit (AIC = 41,825.190, BIC = 42,251.675, SABIC = 41,997.535) compared with Bi-HM (AIC = 41,969.750, BIC = 42,289.613, SABIC = 42,099.099) and revealed an inverted U-shaped speed-accuracy pattern. These findings may suggest that modeling nonlinear dependencies can improve the precision of ability estimation in large-scale assessments and inform item design by identifying task features that elicit distinct response processes. The QBi-HM framework offers a flexible tool for researchers and practitioners seeking to leverage response time data to better understand test-taking behavior.

## 1. Introduction

Early psychometric research concentrated on item responses obtained from paper-and-pencil tests; the advent of computer-based administration, however, has fundamentally widened the range of data accessible for psychometric inference. Contemporary large-scale assessments such as PISA and TIMSS now routinely capture process variables—most notably response time—alongside traditional accuracy scores (Liang et al., 2023; van der Linden, 2007; Zhan, 2022). Consequently, response accuracy and response time have become the central pair of outcomes in joint modeling research. The hierarchical modeling (HM) framework proposed by van der Linden (2007) dominates this literature. HM is particularly suited to jointly modelling owing to its principled two-level structure. At the first level, any Item Response Theory (IRT) model may be specified for response accuracy and any Response Time (RT) model for response times, conditional on the latent person parameters. This level models the relationship between observed response time and accuracy and their corresponding item (e.g., difficulty, time intensity) and person parameters (e.g., ability, speed). At the second level, the joint distribution of person and item parameters captures the overall speed–accuracy association through the covariance structure of latent traits, avoiding direct residual dependencies. This decomposition permits the speed–accuracy dependency to be introduced at the latent level—where it is theoretically most meaningful—while preserving conditional independence at the observation level, ensuring computational tractability. Consequently, the HM not only enhances the flexibility of the framework but also provides a solid foundation and paradigm for future research.

Based on the HM framework, subsequent studies have extended this approach from multiple perspectives. Such developments include examinations of the applicability of response time models (Klein Entink et al., 2009), extensions to different test types (Ranger & Ortner, 2012; Zhan, 2022), and applications in psychological experiments (Loeys et al., 2011). Within the HM framework, a key assumption is that response accuracy and time are conditionally independent given these latent person parameters (e.g., ability, speed). However, Bolsinova and Maris (2016) demonstrated that this conditional independence assumption is frequently violated, indicating residual dependencies after accounting for ability and speed. Consequently, researchers have progressively expanded the model from an independent HM to a dependent HM. When modeling this conditional dependency, prior studies have adopted linear and nonlinear approaches. Regarding linear dependencies, Meng et al. (2015) explained the residual correlation between response accuracy and response time based on covariance structures at the item–subject interaction level, although this method constrained the flexible estimation of item parameters. Bolsinova et al. (2017a, 2017b) incorporated residual time into response modeling to capture linear effects of response time on accuracy across both item and person levels. However, their approach tended to overemphasize the role of time. To address these limitations, Guo et al. (2024) proposed a bifactor perspective, treating response accuracy and response time as local factors and their joint effect as a general factor, thereby establishing a Bifactor Hierarchical Model (Bi-HM). Regarding nonlinear dependencies, Chen et al. (2018) controlled for both person and item variability via double centering and identified an inverted U-shaped nonlinear pattern between response time and accuracy across several cognitive tasks, though they did not formalize a quantitative model. Bolsinova and Molenaar (2018) proposed three nonlinear models—quadratic, multi-class, and nonparametric—based on residual time. However, these models still overestimated time-related effects; the multi-class approach relied on subjective categorization, while the nonparametric method was computationally complex. In summary, although existing studies have explored the relationship between response time and accuracy from both linear and nonlinear perspectives within the HM framework, they all exhibit significant limitations. On the one hand, the linear model lacks sufficient descriptive power and fails to capture the nonlinear dynamic pattern in which response time initially rises and subsequently declines as accuracy increases. On the other hand, existing nonlinear models suffer from issues such as overestimation of time effects or excessive model complexity, which constrain their generalizability and practical applicability.

Psychometrics aims to construct appropriate measurement models that establish proper connections between observed item data and latent traits. To capture nonlinear relationships, nonlinear factor analysis incorporates quadratic terms to model curvilinear associations between observed indicators and latent variables (Yalcin & Amemiya, 2001). Rizopoulos and Moustaki (2008) further extended this approach through the generalized latent variable model, which includes quadratic terms to improve the representation of complex latent structures. Similarly, Smits et al. (2016) demonstrated that quadratic factor models not only provide a good approximation to skewed normal factor models but also effectively capture skewness and nonlinearity in the data, offering greater flexibility than conventional skewed normal models. Moreover, Umbach et al. (2017) highlighted that quadratic terms are commonly used in nonlinear structural equation modeling to represent nonlinear effects. In joint modeling of response accuracy and response time, quadratic approaches have also been widely adopted to describe nonlinear relationships. Molenaar et al. (2015) incorporated a quadratic ability term to reveal the nonlinear influence of ability on processing speed. Subsequently, Molenaar and Bolsinova (2017) proposed a quadratic speed factor to capture the speed characteristics of skewed distributions. Bolsinova and Molenaar (2018) directly analyzed the nonlinear effect of residual time on response accuracy using a quadratic term. Collectively, these studies indicate that quadratic modeling is particularly suitable when nonlinear relations exist among latent variables. However, despite attempts to explore nonlinear relationships between response time and accuracy (Bolsinova & Molenaar, 2018), existing methods remain limited. It is worth noting that although Guo et al. (2024) proposed the Bi-HM, which is restricted to linear relationships, its modeling framework offers valuable insights for conceptualizing the dependency between response accuracy and response time.

From a cognitive and behavioral perspective, there are compelling theoretical reasons to expect a nonlinear—specifically inverted U-shaped—relationship between response accuracy and response time in assessment contexts. The speed–accuracy trade-off (SAT) framework posits that individuals continuously adjust their response strategies to balance competing demands for speed and accuracy (Heitz, 2014). This adjustment is not monotonic: at very short response times, accuracy tends to be poor due to insufficient cognitive processing; as time increases, accuracy improves with adequate resource allocation; yet beyond an optimal point, further time investment may yield declining accuracy, reflecting disengagement, fatigue, or inefficient strategies (Chen et al., 2018; Ratcliff & McKoon, 2008). This trajectory aligns with the Yerkes–Dodson law (Yerkes & Dodson, 1908), which posits an optimal arousal level for peak performance, beyond which functioning deteriorates. Evidence accumulation models, such as the diffusion model (Ratcliff & McKoon, 2008), further explain this nonlinearity through the interplay of drift rate, boundary separation, and non-decision time—parameters that produce differential speed–accuracy mappings across tasks and individuals. Empirically, Chen et al. (2018) documented robust inverted U-shaped patterns across multiple cognitive tasks after controlling for person and item effects, confirming that this nonlinearity is a genuine phenomenon rather than a format-specific artifact. These considerations collectively justify incorporating quadratic terms to capture the curvilinear speed–accuracy relationship in psychometric models.

In addition, from the perspective of establishing an appropriately specified psychometric model, capturing the true functional form of the relationship between response accuracy and response time is of central practical importance. Linear dependency models, while parsimonious, impose a monotonic assumption that may systematically distort parameter estimates and misrepresent the underlying cognitive processes when the true relationship is curvilinear (Bolsinova & Molenaar, 2018; Chen et al., 2018). In large-scale educational assessments such as PISA and TIMSS, where test scores directly inform educational policy and resource allocation (Li et al., 2025), model misspecification can lead to biased ability estimates and compromised comparability across administrations (Kreiner & Christensen, 2022). For diagnostic assessment, an incorrectly assumed linear relationship can lead to less accurate person parameter estimates and the decrease in attribute and pattern correct classification rates (Qiao et al., 2021). Moreover, from a test development and validation standpoint, understanding the true speed–accuracy trade-off curve provides valuable guidance for setting appropriate time limits, designing items that elicit optimal processing, and interpreting performance outcomes in a way that is both fair and theoretically defensible. Evaluating and, when necessary, adopting nonlinear specifications in psychometric models is not merely a statistical refinement but a fundamental step toward ensuring the validity, fairness, and practical utility of measurement instruments in real-world assessment contexts. Therefore, to comprehensively and accurately characterize the nonlinear relationship between these two variables, it is necessary to develop a model that integrates the bifactor hierarchical structure with quadratic effects.

To this end, this study incorporates quadratic terms into the linear bifactor hierarchical model (Bi-HM), thereby proposing the Quadratic Bifactor Hierarchical Model (QBi-HM). Compared with Bi-HM, QBi-HM not only remains compatible with standard latent variable modeling software such as Mplus, but also captures nonlinear effects between response accuracy and response time. Its generalizability lies precisely in the fact that it can be estimated using widely available software (e.g., LISREL, AMOS). This study addresses three specific research questions: (1) Can QBi-HM accurately recover model parameters under nonlinear data-generating conditions? (2) Does QBi-HM recover parameters accurately when the true data-generating model is the linear Bi-HM? (3) Does QBi-HM provide superior fit to empirical data compared with Bi-HM and the standard hierarchical model?

## 2. Quadratic Bifactor Hierarchical Model

### 2.1. Model Construction

In QBi-HM, the response accuracy $X_{ij}$ of the examinee *i* (*i* = 1, …, N) to item *j* (*j* = 1, …, m) is modeled using a two-parameter logistic model, which can be expressed as

(1) $$P\left(X_{ij}=1|\theta_{i},\eta_{i}\right)=\frac{exp\left(a_{j}\theta_{i}+\lambda_{11j}\eta_{i}+\lambda_{12j}\eta_{i}^{2}-d_{j}\right)}{1+exp\left(a_{j}\theta_{i}+\lambda_{11j}\eta_{i}+\lambda_{12j}\eta_{i}^{2}-d_{j}\right)}$$

where $a_{j}$ and $d_{j}$ denote the item discrimination parameter and difficulty parameter, respectively; θ represents the local factor for the response accuracy and also represents the ability parameter of the examinee, η indicates the general factor underlying both the examinee’s response accuracy and response times, as well as a parameter capturing the speed–accuracy trade-off; $\eta^{2}$ is the quadratic term of the examinee’s speed-accuracy trade-off parameter, reflecting its quadratic effect on accuracy. $\lambda_{11}$ is the linear effect of the examinee’s speed-accuracy trade-off parameter on item accuracy, and $\lambda_{12}$ is the nonlinear effect of the quadratic term of the examinee’s speed-accuracy trade-off parameter on item accuracy.

For the response time $T_{ij}$ of examinee *i* on item *j*, it is commonly assumed that, after applying a logarithmic transformation, $logT_{ij}$ follows a normal distribution. This model can be expressed as follows:

(2) $${logT}_{ij}\sim Ν\left(\beta_{j}-\tau_{i}+\lambda_{21j}\eta_{i}+\lambda_{22j}\eta_{i}^{2},\;\alpha_{j}^{-2}\right)$$

where $\beta_{j}$ and $\alpha_{j}$ denote the item time intensity parameter and the item time discrimination parameter, respectively; $\tau_{i}$ represents the local factor for the response time data and also represents the speed parameter of the examinee; η and $\eta^{2}$ represent the linear and quadratic terms of the speed–accuracy trade-off parameter, reflecting their linear and quadratic effect on response time; $\lambda_{21}$ is the linear effect of the examinee’s speed-accuracy trade-off parameter on item response time; $\lambda_{22}$ is the nonlinear effect of the quadratic term of the examinee’s speed-accuracy trade-off parameter on item response time.

At the person level, the ability parameter θ, speed parameter τ, and speed–accuracy trade-off parameter η are assumed to be mutually independent, each following a standard normal distribution. For the item discrimination parameters, a and α are assumed to follow independent left-truncated normal distributions with a mean of zero, a variance of one, and a lower bound of zero. The item difficulty parameters and response time intensity parameters are assumed to jointly follow a bivariate normal distribution, as specified in Equation (3):

(3) $$\left[\begin{array}{c} d_{j}\\ \beta_{j} \end{array}\right]\sim MVN\left(\mu_{I}=\left[\begin{array}{cc} \mu_{d} & \mu_{\beta } \end{array}\right],\Sigma_{I}=\left[\begin{array}{cc} \sigma_{d}^{2} & \sigma_{d\beta }\\ \sigma_{d\beta } & \sigma_{\beta }^{2} \end{array}\right]\right)$$

To more purely describe the relationship between response time and accuracy, QBi-HM adopts a bifactor structure in which the general factor η represents the shared speed–accuracy trade-off, while the local factors θ and τ capture domain-specific ability and speed propensities, respectively (Cai et al., 2011; Reise, 2012). Orthogonality constraints among η, θ, and τ ensure that the shared speed–accuracy component is isolated from unique ability and speed tendencies, thereby preventing the confounding of common and specific variance sources (Reise, 2012; Eid et al., 2017). Beyond the linear bifactor decomposition, QBi-HM incorporates a quadratic term η^2^ to capture the nonlinear dynamics of the speed–accuracy relationship. Rather than assuming that additional time investment monotonically improves accuracy, the quadratic specification models an inverted U-shaped trajectory in which moderate deliberation enhances performance, but excessive time may reflect fatigue or inefficient processing, causing accuracy to decline (Chen et al., 2018). This curvilinear pattern aligns with the Yerkes–Dodson law, which posits that performance peaks at intermediate levels of cognitive arousal (Yerkes & Dodson, 1908; Heitz, 2014). Item-level parameters $\lambda_{12}$ and $\lambda_{22}$ govern the curvature of this nonlinear trajectory for each outcome, allowing the model to characterize how task demands differentially shape the optimal speed–accuracy zone.

### 2.2. Relationship Between QBi-HM and Other Models

To clarify the positioning of QBi-HM, it is useful to first consider the standard hierarchical model (HM; van der Linden, 2007). In HM, the dependency between response accuracy and time is represented at the person level by a correlation between the ability factor (θ) and the speed factor (τ). This specification treats the speed–accuracy relationship as linear and uniform across items, with any systematic co-variation between the two outcomes absorbed entirely by this single correlational parameter. Guo et al. (2024) proposed the linear bifactor hierarchical model (Bi-HM) as an alternative decomposition. Rather than allowing θ and τ to correlate, Bi-HM introduces a general factor η that is orthogonal to both θ and τ. Under this orthogonal design, θ captures only the accuracy-specific propensity, τ captures only the time-specific propensity, and η absorbs the shared variance between response time and accuracy. Thus, θ and τ function as local factors representing unique cognitive capacity and baseline processing speed, respectively, whereas η represents the common component that simultaneously influences both outcomes. Psychometrically, η is not merely a residual correlation but a theoretically motivated latent construct: it reflects a trait-like speed–accuracy trade-off (SAT) orientation. The orthogonal structure is critical to this interpretation (Cai et al., 2011; Reise, 2012). Because η is constrained to be uncorrelated with θ and τ, any variance attributable to general cognitive ability—which would otherwise confound η—is deliberately excluded. Consequently, η is defined as that portion of performance which systematically varies with pacing strategy and jointly affects both the probability of a correct response and the observed response time, above and beyond pure ability and speed. In other words, η captures the examinee’s consistent tendency to balance speed against accuracy across items, making it a dedicated SAT factor rather than an all-purpose residual (Heitz, 2014). We emphasize that this characterization is a theoretically motivated interpretation of the orthogonal bifactor decomposition; it does not constitute direct empirical evidence that η measures a cognitive mechanism of speed–accuracy trade-off. QBi-HM builds directly upon this bifactor logic but adds a crucial refinement: the general factor η is permitted to influence response time and accuracy nonlinearly. This latent-level quadratic specification offers three advantages over residual-time nonlinear approaches: (a) it treats response accuracy and response time symmetrically as local factors, avoiding the overemphasis on time that characterizes residual-time models; (b) the orthogonal bifactor structure isolates the shared speed–accuracy component from general cognitive proficiency, preventing confounding; and (c) the parametric quadratic form remains computationally tractable within conventional latent variable software, circumventing the subjective categorization or intensive nonparametric integration required by alternative nonlinear methods (Bolsinova & Molenaar, 2018). Substantive cognitive theories, including the speed–accuracy trade-off framework and evidence accumulation models, suggest that the relationship between processing time and accuracy is often curvilinear rather than monotonic. Performance typically follows an inverted U-shaped pattern, where moderate deliberation enhances accuracy, but excessive time may lead to fatigue or inefficient strategy use, causing accuracy to decline (Chen et al., 2018; Yerkes & Dodson, 1908). By incorporating quadratic effects of η, QBi-HM parameterizes this latent-level curvilinearity, allowing the marginal relationship between response time and accuracy to change as an examinee moves toward extreme speed or extreme accuracy orientations. In addition, the item-level coefficients λ govern how the person-level trait η is expressed across specific items, analogous to how item discrimination parameters moderate ability effects in conventional IRT. These coefficients do not imply that η itself is structurally determined by item characteristics; rather, they characterize item-specific sensitivity to the speed–accuracy trade-off orientation.

In terms of model nesting, QBi-HM reduces to Bi-HM when the nonlinear effects of η are absent (i.e., λ_12_ = λ_22_ = 0). If the linear shared effects are also absent (i.e., λ_11_ = λ_21_ = 0), the model further collapses to the standard HM, provided that the correlation between θ and τ is reintroduced to capture any remaining linear association. It is important to note that whereas Bi-HM and QBi-HM impose orthogonality constraints among all person parameters (θ, τ, and η), the standard HM explicitly characterizes the speed–accuracy relationship through the joint distribution of person parameters—specifically, a nonzero covariance between θ and τ at the second level (van der Linden, 2007). Thus, reverting from the bifactor specification to HM requires restoring this latent covariance structure, ensuring that the linear dependency between response accuracy and time is preserved through correlated latent factors rather than through a shared general factor.

### 2.3. Parameter Estimation and Model Identification

Parameter estimation for both QBi-HM and Bi-HM was conducted using robust maximum likelihood (MLR) estimation in Mplus 8.1 (Muthén & Muthén, 2019). Because QBi-HM includes a quadratic interaction term between latent variables, numerical integration was required; we employed adaptive Gauss-Hermite quadrature with 15 integration points per dimension. Convergence was assessed using the default Mplus criteria: for the quasi-Newton step, the convergence criterion was set to 0.100 × 10^−5^; for the EM algorithm, convergence required that the log-likelihood change be below 0.100 × 10^−2^, the relative log-likelihood change below 0.100 × 10^−5^, and the maximum derivative below 0.100 × 10^−2^. For model identification, the variances of the ability parameter (θ), speed parameter (τ), and speed–accuracy trade-off parameter (η) were fixed at 1, and the means of these parameters were fixed at 0 (Cai et al., 2011; Guo et al., 2024; Reise, 2012). Additionally, consistent with conventional bifactor model specifications, correlations among the three person parameters were constrained to zero (Cai et al., 2011; Reise, 2012). Correlations among item parameters were omitted from the estimation. Mplus does not readily support their inclusion in this framework. Prior evidence indicates that ignoring such correlations has negligible impact on parameter recovery in HM (Molenaar et al., 2015). This simplification is also consistent with extended hierarchical models that omit these relationships (Wang et al., 2013). The corresponding Mplus code is available at https://www.scidb.cn/anonymous/SVpmMkFy (accessed on 19 November 2025).

## 3. Simulation Study

To enhance the representativeness of the simulation studies, response accuracy and response time data were generated using both QBi-HM and Bi-HM as baseline models. This design enabled a systematic evaluation of how different conditions affected parameter estimation accuracy for each model, as well as an assessment of their robustness. Accordingly, two simulation studies were conducted. In Simulation Study 1, data were generated from QBi-HM as the baseline model. This study examined how neglecting the nonlinear relationship between response accuracy and response time affected Bi-HM performance, while also evaluating the parameter estimation accuracy of QBi-HM itself. Simulation Study 2 used Bi-HM as the baseline model for data generation. It focused on investigating how linear dependencies between response accuracy and response time influenced the robustness of QBi-HM, and it assessed the estimation accuracy of Bi-HM parameters.

### 3.1. Evaluation Metrics

All simulation results were assessed using mean squared error (MSE) and average bias. Lower MSE values reflect closer alignment between estimated parameters and simulated values, indicating higher estimation accuracy. Similarly, bias values nearer to zero indicate smaller relative deviations in parameter estimates, representing better estimation performance.

(4) $$MSE\left(\hat{\xi }\right)=\frac{\sum_{r=1}^{R}\sum_{k=1}^{K}{\left(\xi -\hat{\xi }\right)}^{2}}{R*K}$$

(5) $$Bias\left(\hat{\xi }\right)=\frac{\sum_{r=1}^{R}\sum_{k=1}^{K}\left(\xi -\hat{\xi }\right)}{R*K}$$

where $\hat{\xi }$ and ξ represent the estimated and simulated values, respectively; R denotes the number of repetitions; and K represents the number of examinees or test items.

### 3.2. Simulation Study 1

#### 3.2.1. Simulation Conditions and Data Generation Using QBi-HM

A Monte Carlo simulation study was conducted to evaluate the recovery of all model parameters under varying sample sizes and test lengths. The design included two factors: sample size (*N* = 1000, 1500, 3000) (Meng et al., 2015; Bolsinova et al., 2017b) and test length (*m* = 30, 60) (Bolsinova et al., 2017b), resulting in a 3 × 2 fully crossed design with six conditions. Each condition was independently replicated 50 times to obtain stable estimates of bias and mean squared error (MSE) for each parameter.

All data generation and subsequent analyses were performed in R (version 4.5.3) using the MASS package for multivariate normal draws and custom functions for the logistic and normal models. The data generation procedure proceeded as follows. First, for each examinee, the ability parameters (θ), speed parameters (τ), and the speed–accuracy trade-off parameters (η) were independently sampled from a standard normal distribution *N*(0, 1) (Cai et al., 2011; Guo et al., 2024; Reise, 2012). Next, item parameters were generated. The difficulty-related parameters $\left(d_{j}\right)$ and the response time intensity parameters $\left(\beta_{j}\right)$ were jointly drawn from a bivariate normal distribution with mean vector $\mu_{J}=(0,4)$ and $\Sigma_{J}=\left(\begin{array}{cc} 1 & 0.4\\ 0.4 & 1 \end{array}\right)$ (Meng et al., 2015). Item discrimination parameters $\left(a_{j}\right)$ and item time discrimination parameters $\left(\alpha_{j}\right)$ follow left-truncated normal distributions $N\left(0,1\right)I\left(0,+\infty \right)$ (Meng et al., 2015). The linear coefficients $\lambda_{11j}$ are drawn from $N\left(2,1\right)$ and $\lambda_{21j}$ from $N\left(0,1\right)$ (Guo et al., 2024), and the quadratic coefficients $\lambda_{12j}$ and $\lambda_{22j}$ were sampled from $N\left(-0.5,1\right)$ and $N\left(0,1\right)$, respectively. Finally, based on Equations (1) and (2), for each examinee and item, the response accuracy *X*_ij_ was simulated from a Bernoulli distribution with probability given by Equation (1), and the logarithmic response time ${logT}_{ij}$ was drawn from a normal distribution with mean $\beta_{j}-\tau_{i}+\lambda_{21j}\eta_{i}+\lambda_{22j}\eta_{i}^{2}$ and variance $\alpha_{j}^{-2}$. This entire generation process was repeated for every replication and every condition.

#### 3.2.2. Comparison of Parameter Results Based on QBi-HM

All simulation replications converged successfully and without improper solutions. The parameter estimation results for the QBi-HM as the baseline model are shown in Table 1. For the discrimination parameter a, both the MSE and Bias for QBi-HM approached zero across all conditions, with further improvement observed as sample size increased. In contrast, MSE for Bi-HM generally exceeds 0.86, and its Bias ranges from −0.67 to −0.73, indicating substantial overestimation. For the difficulty-related parameter *d*, QBi-HM yielded MSE values between 0.006 and 0.080 and Bias ranging from −0.100 to 0.003, reflecting high estimation accuracy with minimal bias. Conversely, Bi-HM produced MSE values from 0.633 to 0.980 and Bias between −0.475 and −0.389. For the time intensity parameter *β*, MSE and Bias for QBi-HM were both near zero, indicating stable and accurate estimates. Although Bias for Bi-HM also approached zero, its MSE ranged from 0.300 to 0.382, which was notably higher than that of QBi-HM. Regarding the time discrimination parameter α, QBi-HM achieved MSE and Bias values close to zero, whereas Bi-HM exhibited Bias between 0.116 and 0.140, demonstrating clear systematic bias, along with a significantly higher MSE compared to QBi-HM. For the linear speed–accuracy trade-off parameters $\lambda_{11}$ and $\lambda_{21}$, QBi-HM produced MSE and Bias values both below 0.12. In comparison, Bi-HM showed an MSE ranging from 3.068 to 3.857 and Bias between 1.238 and 1.357, indicating severe underestimation. Finally, for the quadratic speed–accuracy trade-off parameters $\lambda_{12}$ and $\lambda_{22}$, QBi-HM displayed MSE and Bias values close to zero, demonstrating excellent estimation stability and accuracy.

Table 2 presents the comparison results of examinee parameter estimates across different models, using QBi-HM as the baseline model. As the number of test items increased, QBi-HM consistently attains higher estimation accuracy than Bi-HM. Specifically, when test length increased from 30 to 60 items, QBi-HM showed a marked reduction in MSE for all examinee parameters. MSE for ability (θ) decreased from 0.311 to 0.157, and for the speed–accuracy trade-off parameter (η) from 0.134 to 0.021. In contrast, although Bi-HM performed similarly to QBi-HM in estimating the speed parameter (τ), it exhibited consistently higher MSE for both ability and speed–accuracy trade-off parameters. These results suggest that increasing test length improves both the accuracy and stability of examinee parameter estimation under QBi-HM.

### 3.3. Simulation Study 2

#### 3.3.1. Simulation Conditions and Data Generation Using Bi-HM

In Simulation Study 2, using the same experimental conditions as Simulation Study 1, we manipulated two factors: sample size (*N* = 1000, 1500, 3000) and test length (*m* = 30, 60). This yielded a 3 × 2 fully crossed factorial design with six conditions, each replicated 50 times. MSE and bias were computed separately for each parameter.

During data generation, examinee parameters were generated first. Ability parameter (θ), speed parameter (τ), and speed-accuracy trade-off parameter (η) were all sampled from a standard normal distribution N(0,1). Next, item parameters were generated. The mean vector for the difficulty parameter $d_{j}$ and time intensity parameter $\beta_{j}$ was $\mu_{I}=(0,4)$. The covariance matrix was specified with variances $\sigma_{d}^{2}=\sigma_{\beta }^{2}=1$ and a fixed covariance $\sigma_{d\beta }=0.4$. Values of $d_{j}$ and $\beta_{j}$ for each item were generated according to Equation (3). Item discrimination parameters $a_{j}$ and item time discrimination parameters $\alpha_{j}$ followed a left-truncated normal distribution $N\left(0,1\right)I\left(0,+\infty \right)$. The linear item-level speed-accuracy trade-off parameters $\lambda_{11j}$ were drawn from $N\left(2,1\right)$ and $\lambda_{21j}$ from $N\left(0,1\right)$. Finally, based on the Bi-HM, each examinee’s item response accuracy X_ij_ and logarithmic response time ${logT}_{ij}$ were generated.

#### 3.3.2. Comparison of Parameter Results Based on Bi-HM

All simulation replications converged successfully and without improper solutions. Table 3 presents the parameter recovery results for each model parameter under the Bi-HM baseline. As sample size increased from 1000 to 3000, both QBi-HM and Bi-HM exhibited similar MSE values that gradually decreased across all item parameters, with bias remaining close to zero. For the item discrimination parameter a, MSE decreased from 0.018 to 0.005 in both models. Regarding the difficulty parameter d, MSE decreased from 0.023 to 0.007 under QBi-HM and from 0.017 to 0.005 under Bi-HM. For the linear speed–accuracy trade-off parameter $\lambda_{11}$, MSE decreased from 0.041 to 0.012 under QBi-HM and from 0.032 to 0.009 under Bi-HM. Both models produced comparable recovery for $\lambda_{21}$.

Table 4 presents the estimation results for examinee parameters under each model. Using Bi-HM as the baseline, both QBi-HM and Bi-HM showed comparable estimation accuracy across all examinee parameters. Each model yielded similarly low MSE and Bias value, with Bias generally close to zero. As test length increased from 30 to 60 items, MSE for each examinee parameter in both models decreased steadily. For ability parameter θ, MSE decreased from 0.289 to 0.173 under QBi-HM and from 0.288 to 0.172 under Bi-HM; For speed parameter τ, MSE decreased from 0.030 to 0.015 under QBi-HM and from 0.029 to 0.014 under Bi-HM. For the speed-accuracy trade-off parameter η, MSE decreased from 0.062 to 0.032 under QBi-HM and from 0.060 to 0.031under Bi-HM.

### 3.4. Summary

The simulation results indicate that QBi-HM outperformed Bi-HM in estimating both examinee and item parameters when data were generated from QBi-HM or when nonlinear dependence between response accuracy and response time was present. Under these conditions, Bi-HM yielded substantially biased estimates. By comparison, when Bi-HM served as the baseline model or linear dependence was assumed, the two models achieved comparable accuracy and precision. Overall, QBi-HM accurately captured the nonlinear relationship when present and, when the true relationship was linear (data generated from Bi-HM), correctly recovered the quadratic effects as zero—consistent with the fact that Bi-HM is nested within QBi-HM.

## 4. Empirical Data Analysis

### 4.1. Data Description

The empirical analysis drew on data from the computer-based mathematics assessment of PISA 2012. The initial dataset covered 32 countries and regions, from which four economies—Shanghai (China), the United States, Singapore, and the Slovak Republic—were selected for focused investigation. The original sample comprised 1754 examinees (Tian et al., 2023). To ensure data completeness, we applied listwise deletion to remove all cases with any missing responses, yielding a final analytical sample of 1527 examinees with complete data on 10 valid test items. The sample distribution is as follows: Shanghai (*n* = 375), Singapore (*n* = 411), the Slovak Republic (*n* = 364), and the United States (*n* = 377).

### 4.2. Model Specification and Comparison

Three hierarchical models were fitted to the empirical data: the hierarchical model (HM; van der Linden, 2007), in which the ability and speed latent factors are assumed to be correlated, the bifactor hierarchical model (Bi-HM; Guo et al., 2024), and the proposed quadratic bifactor hierarchical model (QBi-HM). Model fit was assessed using four indices: log-likelihood (LL), Akaike information criterion (AIC), Bayesian information criterion (BIC), and sample-size-adjusted BIC (SABIC). Higher LL values indicate better model fit, whereas lower AIC, BIC, and SABIC values indicate superior fit and greater parsimony.

### 4.3. Results

Model fitting results are presented in Table 5. AIC, BIC, and SABIC values for HM were consistently higher than those for Bi-HM, and the likelihood ratio (LR) test was statistically significant. Likewise, AIC, BIC, and SABIC values for Bi-HM were higher than those for QBi-HM, with the LR test again reaching significance. These results indicate that incorporating a linear dependency between response accuracy and response time improved model fit relative to assuming independence. Furthermore, modeling nonlinear dependency captured the data characteristics better than a linear relationship, providing the best overall fit to the empirical data.

**Figure 1:**
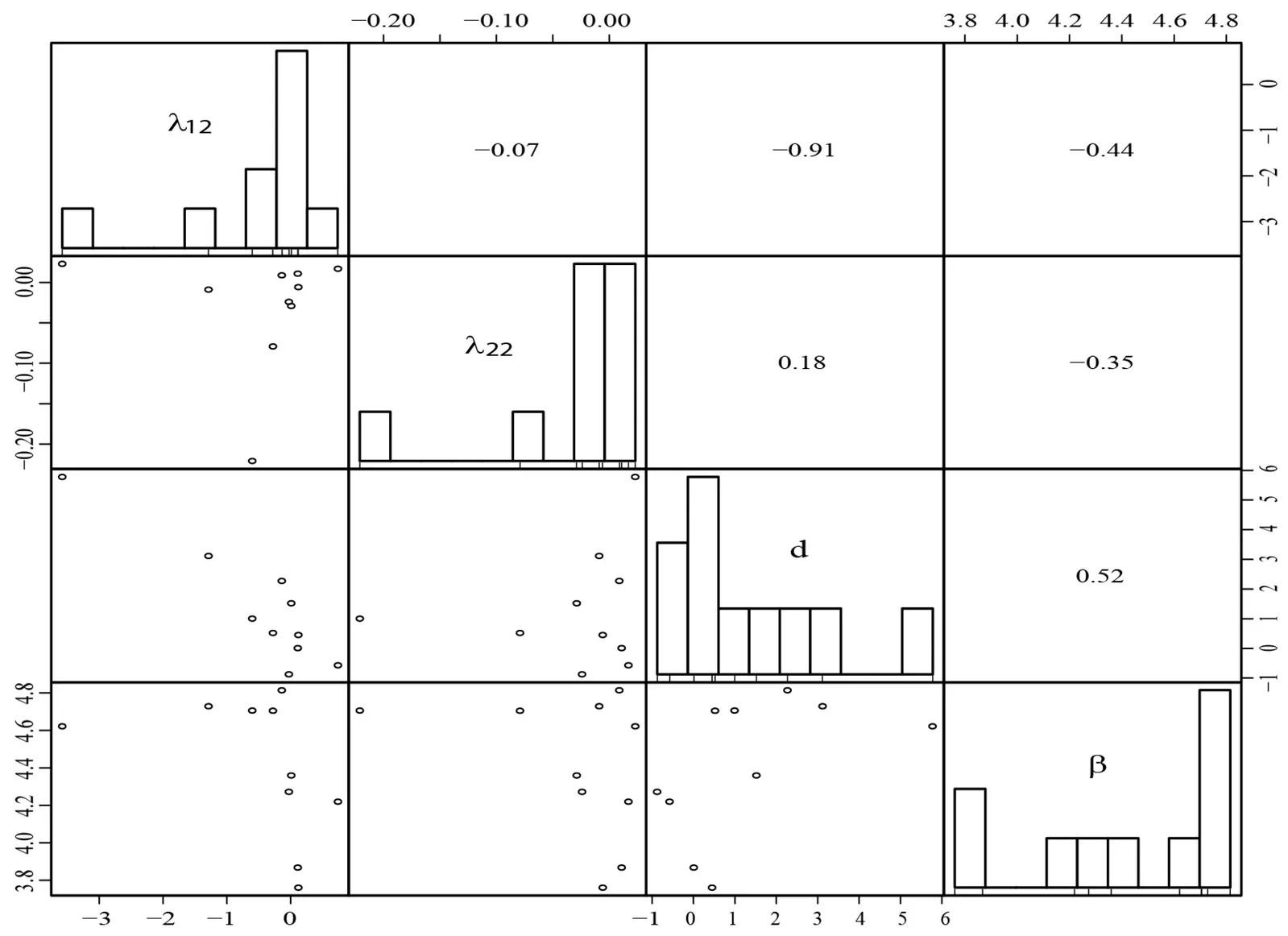
Correlation Matrix of QBi-HM Item Parameters (m = 10 item). The diagonal panels show histograms of the distributions for each parameter: $\lambda_{12}$ (the nonlinear effect of the speed–accuracy trade-off on response accuracy), $\lambda_{22}$ (the nonlinear effect of the speed–accuracy trade-off on response time), d (item difficulty), and β (time intensity). The lower triangular panels display scatter plots of pairwise relationships among these parameters. The upper triangular panels report the corresponding Pearson correlation coefficients.

The correlation matrix of QBi-HM parameters revealed that the speed–accuracy trade-off nonlinearly influenced both response accuracy and response time, with these effects varying by item characteristics (see Figure 1). The quadratic coefficients of the QBi-HM reveal how the speed–accuracy trade-off nonlinearly shapes examinee performance in item-specific ways. The accuracy quadratic coefficient $\lambda_{12}$ correlated strongly and negatively with item difficulty d (r = −0.91) and moderately and negatively with time intensity β (r = −0.44). In practical testing situations, this indicates that on already difficult or time-intensive items, any increase in the general trade-off factor η—reflecting a shift toward faster responding—triggers an accelerating decline in the probability of a correct answer. Because the quadratic term $\lambda_{12}\eta^{2}$ is negative and grows in magnitude as $\left|\eta \right|$ increases, a student who speeds up on a challenging, time-intensive item suffers a disproportionate accuracy penalty that far exceeds what a linear relationship would predict, effectively amplifying the item’s difficulty under time pressure. From an assessment design perspective, items that combine high difficulty with high time intensity are particularly vulnerable to this nonlinear suppression; over-reliance on such items risks introducing construct-irrelevant variance that undermines score interpretability. In contrast, the time quadratic coefficient $\lambda_{22}$ showed only a weak positive correlation with difficulty (r = 0.18) and a moderate negative correlation with time intensity (r = −0.35), suggesting that while time-intensive items modestly dampen the quadratic effect on response times, the nonlinear distortion of speed remains limited overall. Notably, $\lambda_{12}$ and $\lambda_{22}$ were essentially uncorrelated (r = −0.07), indicating that the nonlinear impacts on accuracy and on response time may be follow descriptively different patterns. In educational practice, this implies that helping students regulate their pacing may improve timing anomalies but will not automatically alleviate the steep accuracy losses on difficult items; each dimension requires its own targeted intervention. Consequently, test developers can separately tune item selection and time-limit policies to address the specific nonlinear threats to validity in each dimension.

## 5. Discussion

To advance the joint modeling of response accuracy and response time, this study proposes the Quadratic Bifactor Hierarchical Model (QBi-HM). By extending the Bifactor Hierarchical Model (Bi-HM; Guo et al., 2024) with quadratic terms, QBi-HM captures nonlinear speed–accuracy trade-offs that existing linear specifications cannot accommodate. Simulation results demonstrated that when data were generated from QBi-HM, Bi-HM exhibited substantial systematic bias, whereas QBi-HM achieved markedly higher estimation precision. Conversely, when Bi-HM served as the data-generating model, parameter estimates from both models were broadly consistent. This asymmetry arises because fitting QBi-HM to linear data merely estimates quadratic coefficients near zero without introducing meaningful bias, whereas fitting Bi-HM to nonlinear data omits genuine quadratic effects, resulting in severe systematic bias (Greene, 2018). Empirical analyses further showed that QBi-HM outperformed Bi-HM in model fit, and Bi-HM in turn outperformed the standard HM. These findings suggest that QBi-HM provides a statistically better representation of the observed speed-accuracy relationship. However, as with any relative fit comparison, improved AIC/BIC values indicate superior parsimony and likelihood but do not by themselves confirm cognitive mechanisms. Interpretation of the quadratic coefficients as reflecting distinct processing pathways requires additional validation. From a theoretical standpoint, the bifactor architecture of QBi-HM offers a conceptual parallel to Process Overlap Theory (POT; Kovacs & Conway, 2016), which posits that the positive manifold among cognitive tasks emerges not from a unitary general ability factor but from the overlapping activation of domain-general and domain-specific processes during task performance. In QBi-HM, the general factor η captures the shared speed–accuracy trade-off that modulates both response outcomes, while the local factors θ and τ represent process-specific components tied to accuracy and speed, respectively—mirroring POT’s distinction between shared and specific processes. Furthermore, POT emphasizes that task characteristics determine which processes overlap and how they interact; consistent with this view, our empirical results show that item difficulty and time intensity differentially shape the linear and nonlinear effects of η on accuracy versus time, suggesting that distinct pathways of process overlap are activated depending on task demands. The inverted U-shaped trajectory between speed and accuracy observed in our empirical analysis can also be interpreted through the POT lens as reflecting an optimal zone of process overlap, wherein moderate integration of cognitive processes yields peak processing efficiency, whereas insufficient or excessive overlap impairs performance—a pattern conceptually aligned with the classic Yerkes–Dodson law (Yerkes & Dodson, 1908). By grounding QBi-HM in POT, the present study offers psychometric evidence that nonlinear speed–accuracy trade-offs may reflect fundamental dynamics of cognitive process overlap under varying task conditions, without invoking a single underlying intelligence entity. Moreover, the nonlinear effects of the speed–accuracy trade-off on response accuracy and response time may operate through distinct pathways in shaping examinee response behavior. This differential activation of processing pathways may reflect distinct mechanisms governing accuracy and response time, as well as the different cognitive processes underlying each outcome (Ratcliff et al., 2015). Individual differences in speed–accuracy trade-offs likely modulate these pathways differently. The observed nonlinear effects emerge from the relative contributions of item parameters to each behavioral outcome (Stafford et al., 2020).

Beyond statistical advantages, the QBi-HM may offer substantial practical value by parameterizing item-level linear and nonlinear effects of the speed–accuracy trade-off, potentially enabling test developers to flag items that may be excessively distorted by time pressure—especially high-difficulty, high-time-intensity items with large negative $\lambda_{12}$ values—and to revise or replace them, potentially strengthening score validity. Its hierarchical structure yields a diagnostically rich profile, separating ability (θ), time-management disposition (τ), and the general trade-off tendency (η), which could allow educators to distinguish knowledge deficits from maladaptive speed–accuracy strategies; a student with adequate ability but an extreme nonlinear trade-off pattern might receive targeted test-taking skill training rather than content remediation. In adaptive testing, item-selection algorithms could incorporate the quadratic coefficients to avoid items that introduce speed-related bias at critical ability levels, resulting in fairer and more precise measurement. Ultimately, the model transforms the speed–accuracy trade-off from a nuisance factor into a construct-relevant source of diagnostic information, enhancing both assessment fairness and the educational utility of test feedback. These applications remain speculative until validated by independent empirical studies and operational testing programs.

The current evidence and interpretation are subject to several constraints that warrant further development and refinement. First, while the present simulations demonstrate satisfactory parameter recovery under correct or approximate model specification, they do not assess robustness to asymmetric, threshold-based, or other non-quadratic dependencies. Future work should therefore expand the simulation design to include shorter test lengths (e.g., m = 10), varying magnitudes of quadratic effects, non-normal latent distributions, correlated person and item parameters, missing data, response-time contamination, and alternative nonlinear functional forms. In addition, a more comprehensive set of evaluation metrics—including coverage probabilities, standard-error bias, Type I error rates, and power to detect quadratic effects—should be systematically reported. Furthermore, more flexible functional forms warrant consideration. Although quadratic terms adequately capture symmetric nonlinearities, empirical data may exhibit asymmetric relationships between response accuracy and response time. Semi-parametric approaches (Bolsinova & Molenaar, 2018) could accommodate such complexity without sacrificing computational tractability, offering a promising direction when the underlying functional form is unknown. In addition, the QBi-HM analysis was conducted on only a few isolated instances, and additional instances are required to support the generality of the quadratic term; therefore, claims of significant inverted U-shaped relationships remain preliminary. Second, QBi-HM should be extended beyond unidimensional tests to multidimensional assessment frameworks (He & Qi, 2023), thereby evaluating its robustness across diverse measurement contexts. Third, cross-group heterogeneity in nonlinear speed–accuracy trade-off patterns (Kam & Cheung, 2024) deserves attention, as this would inform the development of more precise and equitable psychometric models. Finally, the integration of artificial intelligence with response time data represents a promising avenue for enhancing model flexibility and predictive accuracy. Neural network-based approaches have demonstrated superior capabilities in capturing complex nonlinear interactions between examinees and items, yet their incorporation into joint modeling frameworks of response accuracy and response time remains underexplored in the psychometric literature (Xiong et al., 2026). Unlike traditional statistical models that rely on prespecified functional forms, deep learning architectures can adaptively learn intricate speed–accuracy relationships from empirical data without strong parametric assumptions. Future research could leverage such techniques to improve parameter estimation precision and offer more nuanced insights into test-taking behavior, thereby complementing the current quadratic specification with a data-driven alternative for modeling nonlinear speed–accuracy trade-offs.

## 6. Conclusions

This study presents the Quadratic Bifactor Hierarchical Model (QBi-HM) as an extension of the linear bifactor hierarchical model (Bi-HM). The findings are as follows:

(1) In simulation studies, QBi-HM produced accurate parameter estimates under both linear and nonlinear data-generating conditions, whereas Bi-HM yielded substantially biased estimates when the relationship was nonlinear.
(2) In the empirical analysis, QBi-HM achieved superior model fit relative to Bi-HM, with quadratic item parameters exhibiting differential correlational patterns with item difficulty and time intensity.
(3) Accounting for potential nonlinear dependencies between response time and accuracy may be essential for a more comprehensive evaluation of person abilities and item characteristics.

## Figures and Tables

**Table 1:** Recovery of Item Parameters with QBi-HM as the Baseline Model.

Item Parameter	Model	*N* = 1000				*N* = 1500				*N* = 3000			
		*m* = 30		*m* = 60		*m* = 30		*m* = 60		*m* = 30		*m* = 60	
		MSE	Bias	MSE	Bias	MSE	Bias	MSE	Bias	MSE	Bias	MSE	Bias
a	QBi-HM	0.035	−0.057	0.017	−0.017	0.017	−0.037	0.010	−0.002	0.008	−0.020	0.006	−0.011
	Bi-HM	1.009	−0.725	1.101	−0.733	0.983	−0.716	0.989	−0.731	0.900	−0.674	0.861	−0.667
d	QBi-HM	0.080	−0.100	0.025	−0.022	0.037	−0.059	0.013	0.003	0.011	−0.013	0.006	0.001
	Bi-HM	0.720	−0.410	0.944	−0.475	0.707	−0.395	0.964	−0.443	0.633	−0.389	0.980	−0.434
β	QBi-HM	0.009	0.001	0.005	0.005	0.005	−0.001	0.003	0.004	0.002	0.001	0.001	0.003
	Bi-HM	0.308	0.016	0.329	−0.005	0.309	0.015	0.364	0.001	0.300	0.011	0.382	0.002
α	QBi-HM	0.003	0.010	0.001	0.001	0.002	0.009	0.001	0.001	0.001	0.004	0.001	0.001
	Bi-HM	0.045	0.121	0.056	0.139	0.048	0.124	0.054	0.139	0.042	0.116	0.053	0.140
$\lambda_{11}$	QBi-HM	0.093	−0.114	0.051	−0.006	0.053	0.025	0.031	−0.014	0.036	0.064	0.017	−0.001
	Bi-HM	3.696	1.357	3.857	1.281	3.462	1.317	3.611	1.275	3.068	1.238	3.857	1.350
$\lambda_{21}$	QBi-HM	0.008	−0.010	0.004	−0.011	0.004	−0.002	0.002	0.002	0.003	−0.003	0.001	−0.001
	Bi-HM	0.738	−0.042	0.793	0.012	0.680	−0.044	0.785	0.018	0.625	−0.016	0.809	−0.016
$\lambda_{12}$	QBi-HM	0.120	−0.114	0.040	0.005	0.057	−0.063	0.021	0.007	0.041	−0.053	0.013	−0.003
$\lambda_{22}$	QBi-HM	0.007	0.002	0.002	−0.001	0.003	−0.001	0.001	−0.004	0.005	0.002	0.001	−0.001

**Table 2:** Recovery of Examinee Parameters with QBi-HM as the Baseline Model.

Examinee Parameter	Model	*N* = 1000				*N* = 1500				*N* = 3000			
		*m* = 30		*m* = 60		*m* = 30		*m* = 60		*m* = 30		*m* = 60	
		MSE	Bias	MSE	Bias	MSE	Bias	MSE	Bias	MSE	Bias	MSE	Bias
θ	QBi-HM	0.311	−0.008	0.167	−0.010	0.283	−0.003	0.156	0.001	0.272	0.001	0.157	−0.001
	Bi-HM	0.939	−0.008	1.025	−0.016	0.929	−0.003	1.039	0.005	0.928	0.001	1.077	0.005
τ	QBi-HM	0.034	−0.057	0.017	0.001	0.031	0.001	0.016	0.004	0.032	0.001	0.015	0.002
	Bi-HM	0.054	0.001	0.028	0.001	0.052	0.001	0.029	0.004	0.054	0.001	0.027	0.003
η	QBi-HM	0.134	0.001	0.030	−0.001	0.082	−0.005	0.021	−0.001	0.055	0.006	0.021	0.001
	Bi-HM	1.602	−0.002	1.721	−0.005	1.476	0.006	1.611	−0.029	1.372	0.014	1.634	−0.033

**Table 3:** Recovery of Item Parameters with Bi-HM as the Baseline Model.

Item Parameter	Model	*N* = 1000				*N* = 1500				*N* = 3000			
		*m* = 30		*m* = 60		*m* = 30		*m* = 60		*m* = 30		*m* = 60	
		MSE	Bias	MSE	Bias	MSE	Bias	MSE	Bias	MSE	Bias	MSE	Bias
a	QBi-HM	0.018	−0.017	0.016	−0.007	0.012	0.001	0.010	−0.008	0.006	−0.003	0.005	0.001
	Bi-HM	0.018	−0.016	0.015	−0.006	0.012	−0.002	0.010	−0.007	0.006	−0.002	0.005	0.001
d	QBi-HM	0.023	0.002	0.023	−0.007	0.013	−0.002	0.014	−0.002	0.007	−0.002	0.007	0.002
	Bi-HM	0.017	0.001	0.018	−0.002	0.010	0.001	0.011	−0.002	0.005	−0.003	0.005	0.001
β	QBi-HM	0.004	−0.006	0.004	0.009	0.003	−0.003	0.002	0.002	0.001	0.002	0.001	−0.001
	Bi-HM	0.003	0.001	0.003	0.007	0.002	−0.001	0.002	0.001	0.001	−0.002	0.001	0.001
α	QBi-HM	0.002	−0.003	0.001	−0.003	0.001	−0.002	0.001	−0.002	0.001	−0.004	0.001	0.002
	Bi-HM	0.001	−0.002	0.001	−0.002	0.001	−0.001	0.001	−0.001	0.001	−0.001	0.001	0.001
$\lambda_{11}$	QBi-HM	0.041	−0.027	0.037	−0.032	0.025	−0.024	0.024	−0.018	0.013	−0.023	0.012	0.001
	Bi-HM	0.032	−0.005	0.029	−0.015	0.020	−0.004	0.019	−0.007	0.010	−0.015	0.009	−0.001
$\lambda_{21}$	QBi-HM	0.003	0.009	0.002	−0.004	0.002	−0.009	0.002	0.001	0.001	−0.002	0.001	−0.001
	Bi-HM	0.003	0.009	0.002	−0.004	0.002	−0.009	0.002	0.001	0.001	−0.002	0.001	−0.001

**Table 4:** Recovery of Examinee Parameters with Bi-HM as the Baseline Model.

Examinee Parameter	Model	*N* = 1000				*N* = 1500				*N* = 3000			
		*m* = 30		*m* = 60		*m* = 30		*m* = 60		*m* = 30		*m* = 60	
		MSE	Bias	MSE	Bias	MSE	Bias	MSE	Bias	MSE	Bias	MSE	Bias
θ	QBi-HM	0.289	0.007	0.179	0.001	0.292	−0.001	0.177	−0.001	0.285	0.002	0.173	0.004
	Bi-HM	0.288	0.007	0.178	0.001	0.291	−0.001	0.176	−0.001	0.284	0.003	0.172	0.004
τ	QBi-HM	0.030	0.001	0.016	0.008	0.029	0.001	0.015	0.001	0.029	−0.002	0.015	0.001
	Bi-HM	0.029	0.001	0.015	0.008	0.029	0.001	0.015	0.001	0.029	−0.002	0.014	0.001
η	QBi-HM	0.062	−0.006	0.034	0.001	0.060	0.003	0.033	−0.001	0.061	−0.002	0.032	−0.002
	Bi-HM	0.060	−0.006	0.033	0.001	0.059	0.003	0.032	−0.001	0.061	−0.002	0.031	−0.002

**Table 5:** Model Fit Indices for Computerized Mathematics Assessment Data.

Model	NP	AIC	BIC	SABIC	LL	LR
HM	41	42,304.200	42,522.774	42,392.527	−21,111.100	
Bi-HM	60	41,969.750	42,289.613	42,099.099	−20,924.875	372.45 **
QBi-HM	80	41,825.190	42,251.675	41,997.535	−20,832.595	184.56 **

Note. NP = Number of parameters; AIC = Akaike’s information criterion; BIC = Bayesian information criterion; SABIC = Sample-Size-Adjusted BIC; LL = Loglikelihood; LR = Likelihood ratio; ** *p* < 0.01.

## Data Availability

The datasets analyzed during the current study are the publicly available PISA 2012 datasets from the OECD. They can be directly downloaded from the website: www.scidb.cn/detail?dataSetId=c1324306d3eb4555a9df4c9c7065fc94&version=V1 (accessed on 30 August 2022).

## Abbreviations

The following abbreviations are used in this manuscript:

QBi-HM	Quadratic bifactor hierarchical model
Bi-HM	Bifactor hierarchical model
HM	Hierarchical modeling
MLR	Robust maximum likelihood
MSE	Mean squared error
LL	Log-likelihood
AIC	Akaike information criterion
BIC	Bayesian information criterion
SABIC	Sample-size-adjusted BIC
LR	Likelihood ratio
NP	Number of parameters
